# Toward an understanding of the chemical ecology of alternative reproductive tactics in the bulb mite (*Rhizoglyphus robini*)

**DOI:** 10.1186/s12862-021-01956-w

**Published:** 2022-01-08

**Authors:** Adam N. Zeeman, Isabel M. Smallegange, Emily Burdfield Steel, Astrid T. Groot, Kathryn A. Stewart

**Affiliations:** 1 Institute for Biodiversity and Ecosystem Dynamics, University of Amsterdam, Amsterdam, The Netherlands; 2 School of Natural and Environmental Sciences, Newcastle University, Newcastle upon Tyne, UK; 3 Institute of Environmental Sciences, Leiden University, Leiden, The Netherlands

**Keywords:** Alternative reproductive tactics, Chemical ecology, Conditional strategy, Environmentally-cued threshold model, Pheromones, Sexual selection

## Abstract

**Background:**

Under strong sexual selection, certain species evolve distinct intrasexual, alternative reproductive tactics (ARTs). In many cases, ARTs can be viewed as environmentally-cued threshold traits, such that ARTs coexist if their relative fitness alternates over the environmental cue gradient. Surprisingly, the chemical ecology of ARTs has been underexplored in this context. To our knowledge, no prior study has directly quantified pheromone production for ARTs in a male-polymorphic species. Here, we used the bulb mite—in which males are either armed fighters that kill conspecifics, or unarmed scramblers (which have occasionally been observed to induce mating behavior in other males)—as a model system to gain insight into the role of pheromones in the evolutionary maintenance of ARTs. Given that scramblers forgo investment into weaponry, we tested whether scramblers produce higher quantities of the putative female sex-pheromone α-acaridial than fighters, which would improve the fitness of the scrambler phenotype through female mimicry by allowing avoidance of aggression from competitors. To this end, we sampled mites from a rich and a poor nutritional environment and quantified their production of α-acaridial through gas chromatography analysis.

**Results:**

We found a positive relationship between pheromone production and body size, but males exhibited a steeper slope in pheromone production with increasing size than females. Females exhibited a higher average pheromone production than males. We found no significant difference in slope of pheromone production over body size between fighters and scramblers. However, scramblers reached larger body sizes and higher pheromone production than fighters, providing some evidence for a potential female mimic strategy adopted by large scramblers. Pheromone production was significantly higher in mites from the rich nutritional environment than the poor environment.

**Conclusion:**

Further elucidation of pheromone functionality in bulb mites, and additional inter- and intrasexual comparisons of pheromone profiles are needed to determine if the observed intersexual and intrasexual differences in pheromone production are adaptive, if they are a by-product of allometric scaling, or diet-mediated pheromone production under weak selection. We argue chemical ecology offers a novel perspective for research on ARTs and other complex life-history traits.

**Supplementary Information:**

The online version contains supplementary material available at 10.1186/s12862-021-01956-w.

## Background

Animals exhibit various behavioral, morphological and physiological adaptations related to their ability to attract and compete for mates [1]. Many of these adaptations have evolved under strong sexual selection, resulting in distinct, intrasexual Alternative Reproductive Tactics, or ARTs [2]. ARTs are pervasive in nature [e.g. 3–8] and explanations for their maintenance include models of frequency-dependent selection on genetic polymorphisms (i.e., the fitness of each ART depends on the relative frequency of all ARTs in a population; [9]) and models of condition-dependent developmental divergence [4, 10–12]. Predominantly, however, ART expressions are considered threshold traits, wherein one tactic is expressed when an environmental or (environmentally-driven) physiological cue reaches a certain, genetically determined threshold during ontogeny, and the alternative tactic is expressed if this threshold is not reached [e.g., 2, 13, 14]. Importantly, such environment-dependent ARTs can only coexist if the fitness functions of the ARTs cross over the gradient of the environmental cue [15]. A salient cue of ART expression is diet [14, 16, 17], because nutritional quality and quantity dictate the body sizes and resource budgets of developing individuals [18], thereby affecting the potential future mating success of different ARTs [2]. For example, large juvenile males likely have sufficient resources to develop into large adults with morphological structures that can be used as weapons when defending their mate against rival males, whereas small juvenile males are unlikely to be successful during combat, and most likely benefit more by refraining from developing weapons to resort to “sneaking” tactics [19]. Such sneaking tactics have been observed across a wide range of taxa [e.g., 4, 7, 20], and would be particularly successful if small(er) males can additionally conceal themselves from rival, fighter males, for example by mimicking a female [21].

Female-mimic mating strategies are found in a variety of taxa [e.g., ruffs, 22; snakes, 23; marine isopod, 24]. Sometimes mimicking mechanisms are visual [25], but female mimicry also exists in organisms that rely on non-visual cues [e.g., chemical/pheromone cues, 24]. In many animal taxa, pheromones are the primary means of intraspecific communication and mate attraction [26–28]. Interestingly, pheromone profiles can vary between individuals of the same species—both in quantity and composition [29]. This variation is often mediated by environmental factors [30, 31], especially diet [32–39]. For example, diet has been demonstrated to influence pheromone profiles directly via essential pheromone precursors [38], and indirectly, through an increased resource allocation towards pheromone production [35]. Within heterogeneous environments, where food availability is highly variable between individuals, selection may act on divergent diet-mediated pheromone profiles and drive the evolution of different pheromone-based ARTs. For example, well-fed individuals may attempt to attract mates by producing high quantities of pheromone, while poorly fed individuals may attempt to be inconspicuous by producing low pheromone quantities, permitting them to sneak past competitors to gain access to females. Although inconspicuous sneaking, or female mimicry, tactics are known to occur in many species with male ARTs [e.g., 4, 5, 7], in most cases, the role of pheromones in these strategies remains unknown [but see 24].

**Fig. 1:**
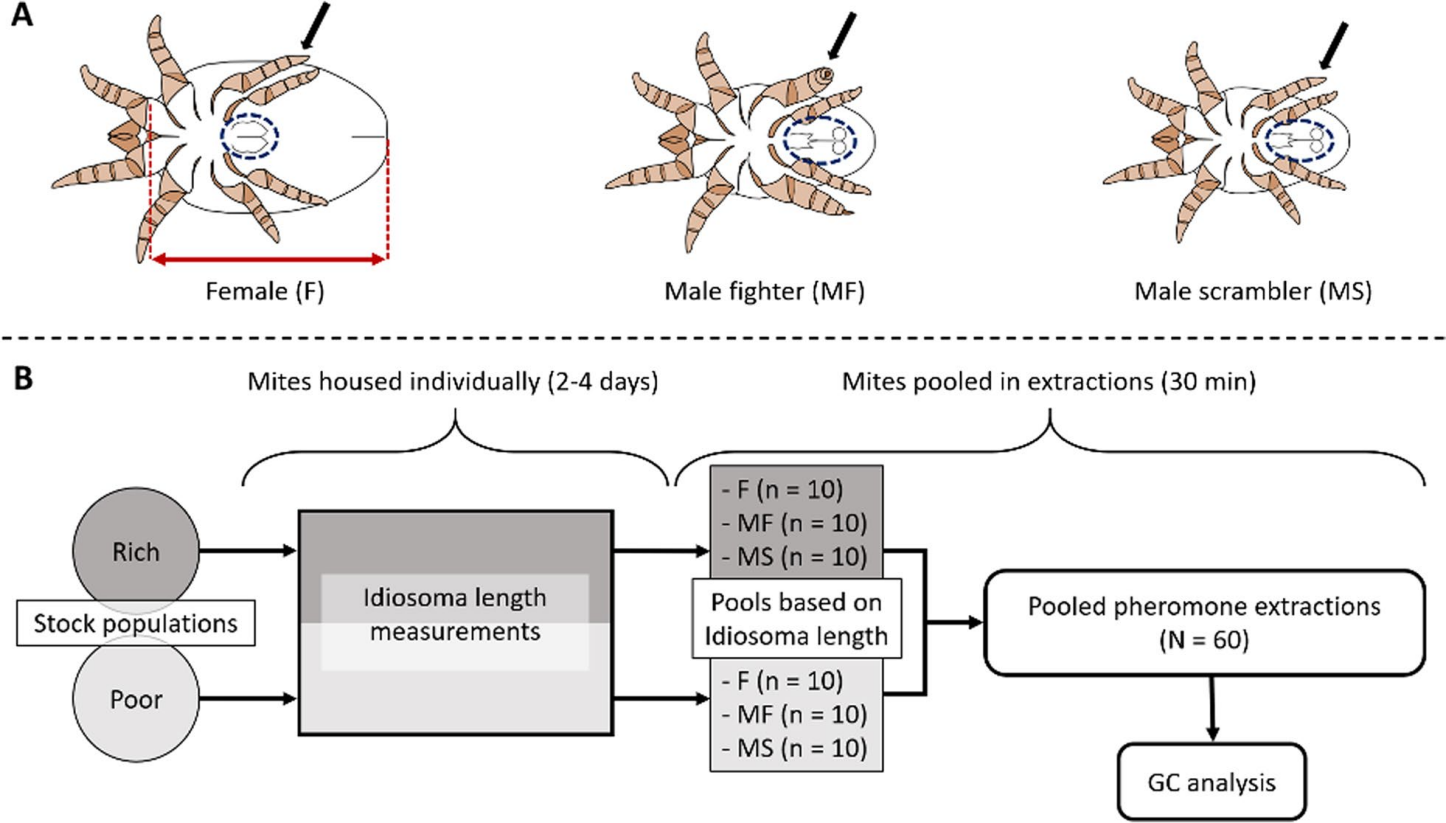
**A** Ventral drawings of adult bulb mites (*Rhizoglyphus robini*). From left to right: female, male fighter and male scrambler. Morphological characteristics useful for identification are highlighted: black arrows indicate the third leg pair (thickened and sharply terminated in fighters), and dotted blue ellipses highlight the genitalia (including the anal discs in the males), which differ distinctly between males and females [42]. The idiosoma length is indicated with a red arrow (between the two dashed red lines; 42, 43) on the drawing of the female. Mite drawings by F. Rhebergen. **B** Workflow for pheromone quantifications: (1) females, male fighters and male scramblers were randomly selected from rich (dark grey) and poor (light grey) stock populations, and housed individually in plastic tubes, (2) the idiosoma length of the collected mites was measured as a proxy for body size, (3) mites from the same stock population and of the same sex or ART, were pooled based on idiosoma length, totaling 60 pools, and (iv) the α-acaridial production of each pool was quantified by performing hexane extractions followed by gas chromatography (GC) analysis

The aim of this study was to gain insight into the importance of pheromones in the maintenance of ARTs. For this, we used the bulb mite (*Rhizoglyphus robini*), a well-studied example of a system in which ART expression is a threshold trait cued by diet. Importantly, ART expression in *R. robini* does not depend on population density, unlike in its sister species *R. echinopus* [40], nor on ART frequency [41]. Upon maturity, male *R. robini* develop into one of two distinct morphs (see Fig. 1A): (1) juvenile males that are relatively large mostly mature as “fighters”, which possess a thickened third leg pair with a sharp end that can be used to kill conspecifics [44, 45], and (2) juvenile males that are relatively small mostly mature as “scramblers”, which lack the weaponized leg pair (although a rare third morph, the mega-scrambler, has recently been suggested; [46]). Although scrambler expression is regulated by a (partially) genetically determined threshold for body size [17, 47, 48] with varying degrees of heritability [47, 49], gene-by-environment interactions also play a key role [50], with temperature [51], and importantly diet quality and quantity thought to be the primary drivers of body size and therefore ART expression [18]. Despite this, the selection pressures that maintain the coexistence of fighters and scramblers are still not fully understood. Numerous studies have attempted to identify or quantify the fitness functions that underlie this evolutionary maintenance [17, 41, 43, 47, 50, 52, 53], but some facets of bulb mite ecology, such as its chemical communication, remain unexplored in this context.

As bulb mites are blind, we would expect them to rely heavily on chemical signals for communication [54]. Currently, a putative female sex-pheromone, α-acaridial [55, 56], has been identified in this species, which can elicit increased mounting behaviour in male bulb mites [56]. Notably, α-acaridial is also produced by males, albeit in lower quantities, suggesting that its female sex pheromone functionality depends on the dosage released from the opisthonotal gland [56]. Interestingly, intrasexual variation in α-acaridial production is greater in bulb mite males than in females, potentially highlighting differential ART investment into the production of this pheromone. Despite this, the role of α-acaridial in the maintenance of fighter-scrambler coexistence is unexplored. For example, because scramblers forgo the development of weaponized legs and spend less energy on aggressive behavior compared to fighters [45], they might invest more into pheromone production. Increased pheromone production may serve to mimic the pheromone profile of the much larger females, ultimately reducing the conspicuousness of scramblers towards competitors and allowing them to avoid costly intrasexual combat. Indeed, scramblers, but not fighters, have occasionally been observed to elicit mating behavior in other males [45, 46], providing some promising evidence for (potentially pheromone-mediated) female mimicry.

Here, we conducted a laboratory experiment to test the hypothesis that bulb mite scramblers produce higher quantities of the female sex pheromone (α-acaridial) than fighters, potentially as a means of avoiding intrasexual reproductive competition. Because nutritional uptake has been shown to be an important driver of ART expression in bulb mites [17, 18], and because diet strongly influences pheromone production across many invertebrates [reviewed by 57], we measured α-acaridial production of mites raised on two diets of different food quality. Also, we included body size as a covariate to control for any body size effects on α-acaridial production. Our results show that (1) α-acaridial production was positively correlated with body size, and this relationship was steeper in males than females, (2) α-acaridial production was influenced by the nutritional environment, (3) on average, females produced more α-acaridial than males, and (4) there was no significant difference in slope of α-acaridial production over body size between the male ARTs.

## Results

### Detection and quantification of α-acaridial

**Fig. 2:**
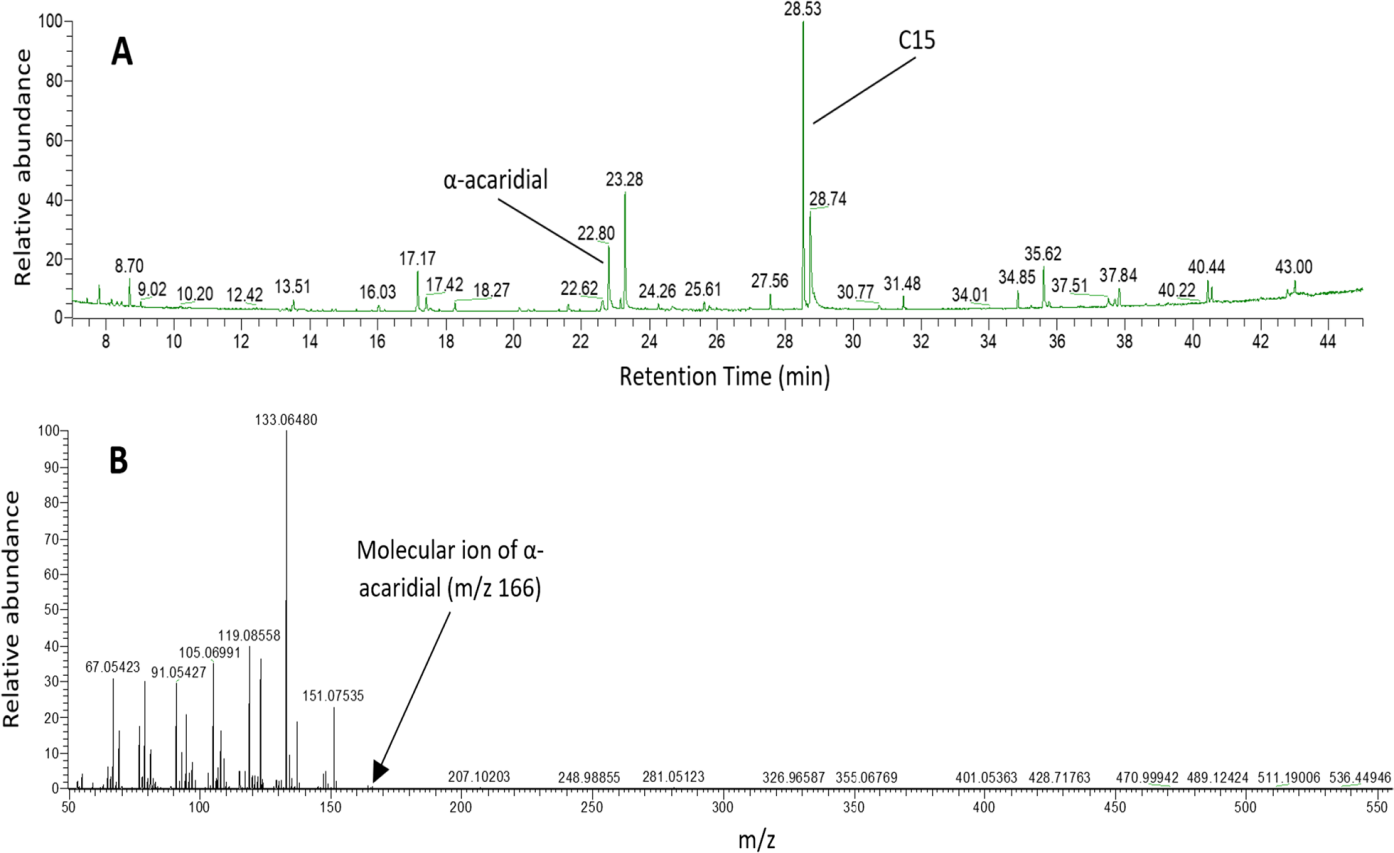
**A** Gas liquid chromatogram of a hexane extract from 10 *Rhizoglyphus robini* females (sampled from the poor stock populations). Numbers above peaks give their retention time. C15; pentadecane (internal standard). **B** Mass spectrum for the peak at 22.8 min. Numbers above peaks give their mass-to-charge ratio (m/z). The ion fragments and the presence of the molecular ion at 166 m/z indicate this compound is α-acaradial [see 55]. See methods for GC–MS conditions

The GC–MS analysis indicated that the pheromone α-acaridial was present in the bulb mite extracts (Fig. 2). Analysis of the ion fragments from the peaks on the GC–MS profiles indicated that the compound at 22.8 min retention time was in fact α-acaridial. The presence of the molecular ion at 166 m/z further supported this [see 55]. Through the GC-analysis, α-acaridial production could be quantified for 56 of the 60 pooled extracts (one outlier was excluded, resulting in N = 55). Thus we had 19 pools of females (n = 8–11 individuals per pool), 18 for fighters, and 18 for scramblers (n = 10–14 individuals per pool), split across the two diet types.

### Effects of morph, diet and idiosoma length on α-acaridial production

**Fig. 3:**
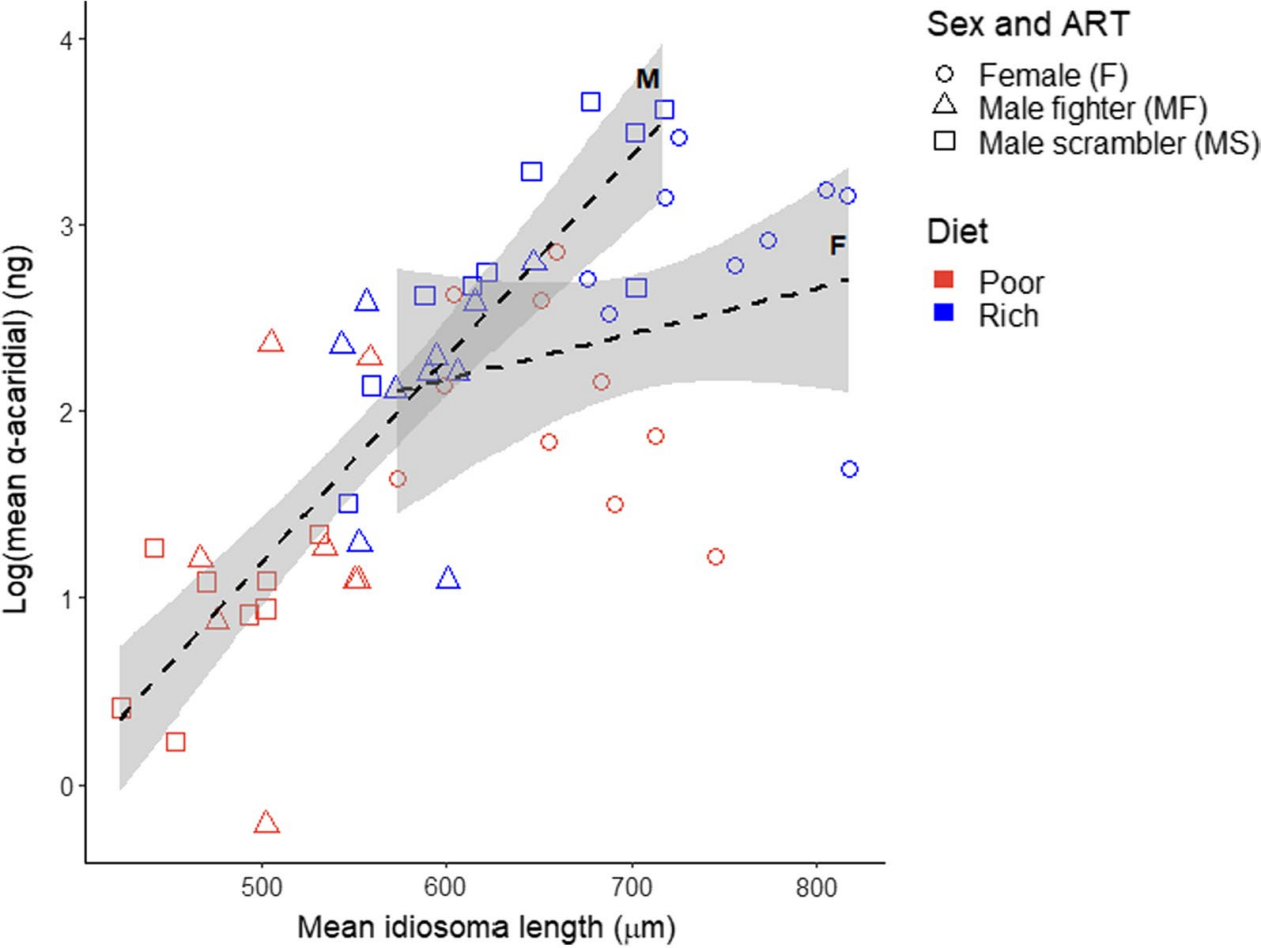
Relationships between idiosoma length and log (α-acaridial production) (shown as per-mite-averages for 55 pools) for bulb mites fed on a rich or poor diet. Illustrated male ARTs grouped together; trendlines for females (F; n = 19) and males (M; n = 36). Standard errors are indicated by grey shading. Legend is shown on the bottom right

**Fig. 4:**
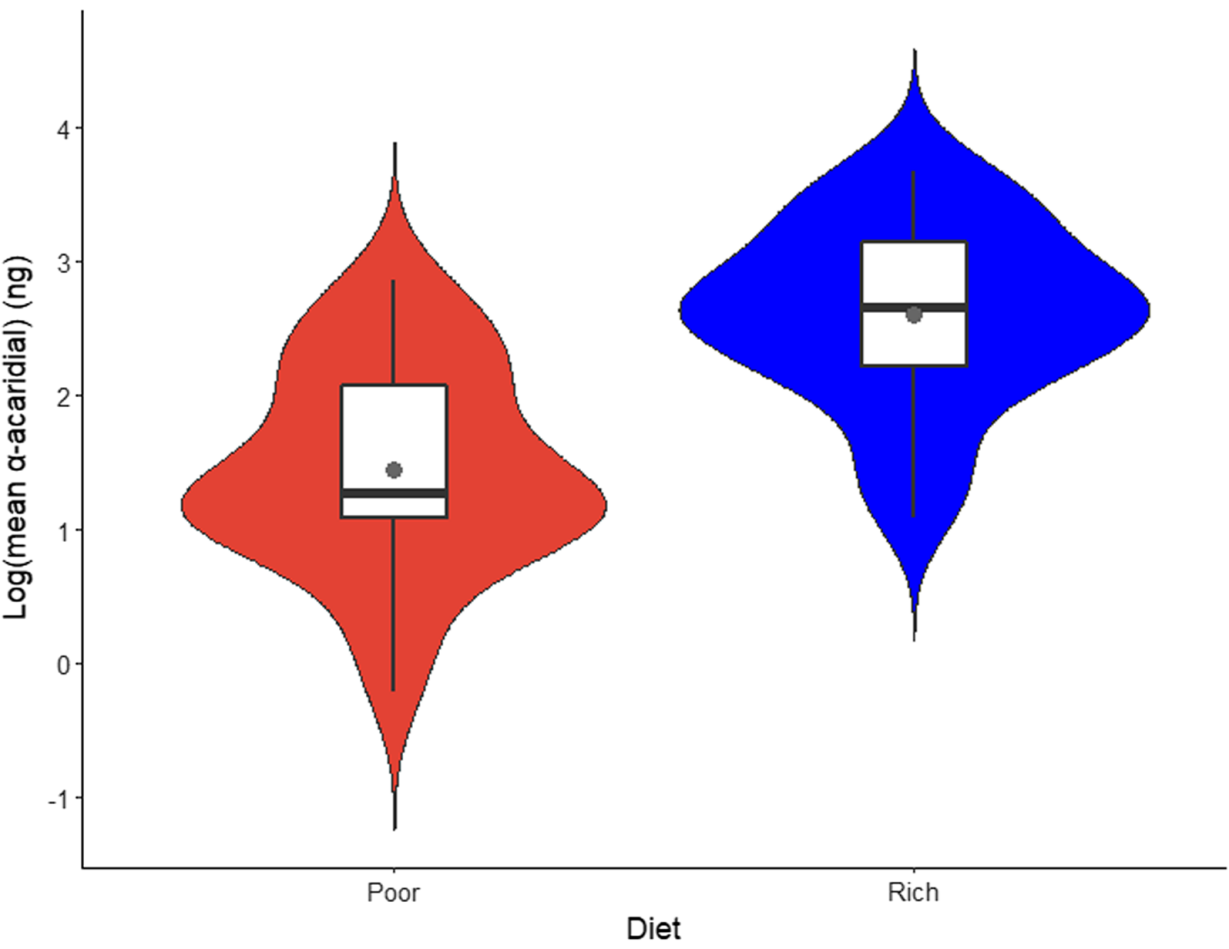
Violin plot, shown as per-mite-averages for 55 pools, of log (α-acaridial production) across nutritional environments for bulb mites fed on a rich (N = 29) or poor (N = 26) diet. Grey dot shows the group mean, black horizontal bar denotes median, boxes represent the interquartile ranges and whiskers show minima and maxima

The stepwise model selection procedure revealed that the relationship between idiosoma length and log (α-acaridial production) differed significantly between the fighters, scramblers and females (Table 1: significant interaction Mo × IL). Merging different, pairwise combinations of fighters, scramblers and females, we found that log (α-acaridial production) of fighters and scramblers did not differ significantly (*F*_1,50_ = 1.283, *p* = 0.287), from which we can infer that males of both morphs showed the same, increase in log(α-acaridial production) with increasing idiosoma length, and which was significantly stronger than that shown by females (Fig. 3). Interestingly, the highest log (α-acaridial production) levels are associated with scramblers, which were also of longer idiosoma length than fighters. We also found that log (α-acaridial production) was significantly higher on the rich diet than on the poor diet (Table 1: significant effect of Diet: rich 2.60 ± 0.66 ng, n = 29 pools; poor 1.45 ± 0.76 ng, n = 26 pools) (Fig. 4). Lastly, overall, mites from the poor diet populations were significantly shorter in idiosoma length than mites from the rich diet populations (rich 654.43 ± 86.55 μm, n = 316; poor 551.65 ± 93.55 μm, n = 282; two sample t-test, *t*_596_ = − 13.95, *p* < 0.001).

**Table 1:** Results of the model selection procedure where Mo is the factor morph (with levels female, fighter, scrambler), D is the factor diet (with levels rich, poor), IL is idiosoma length (µm) (N = 55)

Model parameter	*df*	*F*	*p*
D (Diet)	1	11.475	**0.001**
IL (Idiosoma length)	1	0.344	0.560
Mo (Morph)	2	7.690	**0.001**
IL × Mo	2	8.335	**0.001**

*P*-value significance < 0.05 found in bold

## Discussion

In our study, we aimed to gain insight into the role of pheromones in the evolution and maintenance of ARTs by assessing α-acaridial production in the male-dimorphic bulb mite under two nutritional regimes. We tested the hypothesis that scramblers, given that they forgo investment into weaponized legs, can produce higher quantities of α-acaridial than fighters, which would improve the reproductive success of the scrambler phenotype, e.g., through female chemical mimicry. We found that α-acaridial production was positively correlated with body size, and the slope of α-acaridial production over body size was steeper in males than females. In addition, α-acaridial production was influenced by the nutritional environment, as mites on the rich diet produced more pheromone than those on the poor diet. On average, females produced more α-acaridial than males. Differences between male ARTs were not incorporated in the linear model that best described the data (although they were incorporated into the second-best model), indicating that there is no significant difference in slope of α-acaridial production over body size between the male morphs. Nevertheless, scramblers reached larger maximum body sizes and higher maximum α-acaridial production compared to fighters.

### Intersexual and intrasexual differences in pheromone production

We found that females produced significantly more α-acaridial than males, corroborating the intersexual differences in α-acaridial production described by Mizoguchi et al. [56]. Importantly, the slope of pheromone production over body size was steeper in males than females, indicating that intersexual differences in pheromone production are not due solely to size differences between the sexes. Potentially, males may disproportionately benefit from an increased α-acaridial production with increased size. If, for example, pheromone production acts as an honest indication of quality in bulb mites, sexual selection—which is particularly strong in species with male ARTs [2]—may drive well-conditioned males to produce as much pheromone as they can, without incurring high viability costs. Alternatively, the steeper slope of pheromone production over body size for males may result from differences in chemical ecology between large and small males. The largest males, which in this study are represented mostly by scramblers, produced some of the highest pheromone quantities in the dataset, while many of the smallest males in this study produced very low quantities. This provides some evidence for the hypothesized female mimicry strategy adopted by large scramblers. These large, well-conditioned scramblers may be the only males capable of producing enough pheromones to mimic female pheromone profiles, while also reaching body sizes comparable to females. As such, these scramblers may disproportionately benefit from producing high pheromone quantities compared to smaller males. It should be emphasized however, that this study does not provide evidence that all scramblers are female mimics, as the model that best describes the data did not differentiate between male ARTs. Nevertheless, the results of this study do warrant further exploration of a female mimic or “sneaking” strategy in bulb mites, particularly because ‘mega-scramblers’—which sometimes elicit mating behavior from other males—are suggested to be a third ART [46]. Indeed, these mega-scramblers may be the result of sexual selection driving larger scramblers to chemically and physically resemble females. Intriguingly, some of the largest scramblers in this study, which produced higher pheromone quantities than most females in the dataset, exceeded a body size breakpoint for mega-scramblers calculated by Stewart et al. [46], implying these individuals may in fact represent the mega-scrambler trimorphism.

### Role of nutritional environment and body size in pheromone production

The observed effects of body size and diet both suggest that pheromone production is linked to nutritional uptake—particularly because body size was also dependent on the nutritional environment. The positive correlation between body size and pheromone production may stem from covariation of these variables with diet quality and uptake. As such, individuals that can consume more high-quality food subsequently grow larger [17], while simultaneously maintaining a good enough condition to produce high pheromone quantities. In this context, pheromone production could be a form of honest signaling of *short-term* individual quality [57] particularly for (generally poorer quality) scramblers who may have experienced poor environments during adult metamorphosis but not during mating periods (e.g., somatic buffering [19]). Alternatively, larger individuals may simply produce more pheromone because they have bigger or better developed pheromone glands, and as such, the observed patterns in pheromone production may be non-adaptive. Indeed, there is some support for allometric scaling of the production of defensive chemicals in astigmatic mites, as a study on *Archegozetes longisetosus* showed that the quantity of defensive chemicals from opisthonotal glands scales allometrically with body mass during ontogeny [58]. However, it is not known if this allometric relationship also holds for adult mites with variable mass or body size. Studies on the relationship between body size and pheromone production in other invertebrates have yielded inconsistent results, as positive correlations were found in some species [59–62] while correlations were absent in others [34, 63, but see 59]. Regardless of what drives the observed correlation between body size and pheromone production, the results of this study provide further evidence that the nutritional environment is an important driver of pheromone production, at least in bulb mites. This provides some support for the hypothesis that pheromones are energetically demanding to produce in this species. High nutritional intake likely allows for increased allocation of resources towards pheromone production, which is common among invertebrates [reviewed by 57]. And while it remains unclear to date how sex pheromones in arthropods are largely biosynthesized, evidence suggests acquisition through diet or endosymbionts rather than de novo [e.g., 64], further aligning with the body size and nutritional environment patterns shown here.

### From the chemical ecology of bulb mites to the evolution and maintenance of ARTs; current knowledge and future directions

To better understand the ecological significance of divergent pheromone profiles between sexes and (potentially) male ARTs in bulb mites, it is imperative that pheromone functionality is further elucidated in this species. So far, functionality has only been described for two bulb mite exudates; α-acaridial as a putative female sex pheromone [56] and neryl formate as an alarm pheromone [65]. As a putative sex pheromone, α-acaridial was a good candidate to investigate the role of pheromones in the maintenance of bulb mite ARTs. However, evidence for α-acaridial as a sex pheromone is limited to a single study, where the compound was found to be present in the fractions of female hexane extracts that triggered mounting behavior in males [56]. Synthetic α-acaridial was also shown to elicit mounting behavior at a dose of 10 ng. However, the fact that α-acaridial was also found in males, at quantities that greatly exceed the active dose ([56] reported an average quantity of 163 ± 97 ng for males), indicates a broader function of this compound than a ‘female sex pheromone’. Other bulb mite exudates with potential relevance to the chemical ecology of this species include neral [65], several hydrocarbons [66] robinal, perillene and isopiperitenone [67]. Several of these compounds have also been found in other astigmatic mites [68], but their functionality is mostly unknown in these species.

The lack of knowledge on bulb mite chemical ecology means that there are many avenues for research into pheromone functionality in this species to determine if the intersexual and (to a lesser extent) intrasexual patterns in pheromone production, observed here and by Mizoguchi et al. [56], are adaptive or merely a byproduct of allometric scaling or other factors. Pheromone functions could be elucidated by exposing mites in various ecological settings to the different compounds found in bulb mite extracts [55, 65–67], including different combinations and concentrations to test for synergistic or antagonistic effects. Given the apparent importance of diet in pheromone production observed in this study, further research on how the nutritional environment mediates (e.g., via biosynthesis) pheromone production in bulb mites is warranted. For example, it is unknown to what extent adult pheromone production is driven by nutritional uptake before maturation. In some mite species, juveniles lose the contents of their glands during each molt [69], suggesting that they may be incapable of storing pheromones or other chemicals through their development. Studies on other invertebrates have shown that adult diet, but not diet during the larval stage, affected pheromone production [34] and another found that a rich adult diet can even compensate for the effects of a poor diet during ontogeny [39]. Thus, our study should represent a springboard for myriad future investigations into the role of chemical ecology on the maintenance of ARTs.

Extending beyond mites, other promising model systems for studying the link between chemical ecology and ART maintenance include species where sneaking fertilization occurs from female-like male ARTs that lack anal glands and thus a putative sex pheromone [70], and another species that display pheromonally inconspicuous ejaculate that reduce aggressive male-male interactions [71, 72]. The examples outlined above suggest that pheromones likely play a key role in the success of ARTs across many species, especially when considering the prominent role of pheromones in intraspecific (sexual) communication throughout nature [26–28]. Therefore, future research on ARTs would benefit from comparing pheromone profiles between male ARTs and females (i.e., to explore pheromone based female mimicry) in a variety of taxa.

The direction of putative evolutionary relationships between ARTs and within-population variation in pheromone profiles is another area bearing investigation. So far, we have briefly speculated on how sexual selection may act disruptively on pheromone profiles in heterogeneous environments, and how this may promote the evolution of different pheromone-based ARTs. However, evidence for disruptive selection on pheromone profiles in natural populations is rare [73]. Instead, pheromone profiles and other forms of sexual communication are often under stabilizing selection [74–77]. Therefore, the evolutionary relationship between ARTs and divergent pheromone profiles may be reversed, such that the evolution of ARTs *facilitates* the evolution of divergent pheromone profiles. By definition, ARTs adopt different strategies to improve their reproductive output, and therefore they face different selection pressures [2]. For example, large males that compete for females directly will likely be favored by selection to develop traits that improve their ability to fend off competitors, while smaller males that adopt sneaking tactics may well be favored to develop traits that make them inconspicuous towards other males. These divergent selective pressures may decouple male (sex) pheromone profiles in the population from stabilizing selection, or rather, stabilizing or directional selection may now occur more or less independently for the pheromone profiles of both ARTs, leading to disruptive selection on the population level. There is also emerging evidence that variation in sex pheromone profiles can be maintained by balancing selection [78, 79], e.g., through heterozygote advantage [78]. Thus, within-population variation in pheromone profiles may arise and be maintained through various mechanisms. Further research on the chemical ecology of species with ARTs is needed to assess if divergent pheromone profiles within populations facilitate the evolution of ARTs or vice versa.

## Conclusions

We found a positive relationship between pheromone (α-acaridial) production and body size in bulb mites, but importantly, males demonstrated a steeper slope in pheromone production with increasing size than females. We found no significant difference in slope of pheromone production over body size between fighters and scramblers, but scramblers reached larger maximum body sizes and thus had higher maximum pheromone production compared to fighters. The results of this study also indicate that diet quality influences pheromone production in bulb mites, further highlighting the importance of the nutritional environment for several aspects of the ecology of species displaying environmentally-cued ARTs. The observed patterns of intersexual and intrasexual differences in pheromone production may be adaptive, as sexual selection may have driven the evolution of divergent pheromone profiles that relate to different, condition-dependent strategies, such as sneaking in males. The observed patterns may also be non-adaptive however, potentially reflecting allometric scaling of pheromone production with body size, or diet-mediated pheromone production under weak selection. Further elucidation of pheromone functionality in bulb mites, and additional inter- and intrasexual comparisons of pheromone profiles, are needed to assess the role of pheromones in the maintenance of male-polymorphism in this species.

To our knowledge, this is the first study to directly quantify the production of a pheromone for two ARTs in a male polymorphic species. Yet, intrasexual differences in pheromone production in male-polymorphic species offer promising research avenues in the context of crossing fitness functions that underlie the maintenance of these polymorphisms. Importantly, a more complete understanding of complex life-history traits, such as ARTs, requires investigation through interdisciplinary contexts, such as eco-evolutionary dynamics, developmental biology, population genetics and indeed, chemical ecology.

## Methods

### The bulb mite

The blind bulb mite (*Rhizoglyphus robini*), a common agricultural pest that feeds on various crops [80], is an excellent model system for studying the expression and maintenance of ARTs. In addition to its short generation time and high reproductive output, this microscopic mite can easily be reared in the laboratory under various conditions [54]. After hatching, bulb mites undergo four or five developmental stages: larva, protonymph, deutonymph (a facultative dispersal stage that occurs under adverse conditions, such as food or water scarcity), tritonymph and adult [81]. Transitions between these stages occur in the form of a quiescent molting stage. Upon maturity and depending on the nutritional environment, male bulb mites develop into either armed fighters, or benign scramblers (Fig. 1A).

### Maintenance of stock populations

Stock cultures originated from flower fields near Anna Paulowna (The Netherlands), where they were collected from flower bulbs in 2010. Up until the COVID-19 outbreak in March 2020, stock cultures were kept in an unlit climate chamber (25 ± 1 °C, 60% relative humidity) at the Institute for Biodiversity and Ecosystem Dynamics at the University of Amsterdam (The Netherlands). After that, they were moved to a location without access to climate chambers and kept at room temperature. The experiments were conducted at room temperature as well. Mites were kept in sealed but ventilated plastic containers (50 mm high, 85 mm in diameter) that contained a layer of plaster of Paris (~ 15 mm thick) that was nearly saturated with water. The stock cultures were either always given *ad lib* access to dried yeast granules (Bruggeman instant yeast), or *ad lib* access to grains of rolled oats. Yeast and oats are of high and low nutritional quality, respectively, due to their respective high and low protein content [50]. Therefore, these resources will further be referred to as “rich” (yeast) and “poor” (oats) diets. To reduce inbreeding, stock populations fed on the same diet were intermixed periodically, effectively creating multiple meta-populations. Additional food and water were provided to each stock container once or twice per week (in a manner similar to [43]). The observed heterozygosity (averaged across sex and ART) of the stock populations was measured at 0.39 for the rich environment and 0.48 for the poor environment [50].

### Experimental setup

#### Sampling stock mites

The mites used in the experimental procedures were all randomly selected from the stock cultures (Fig. 1B). The mites were handled (using a fine brush or a metal probe) and identified under a ZEISS Stemi 508 stereomicroscope; their life-history stage, sex and ART were determined based on their body size, genitalia and on the morphology of their third leg pair (Fig. 1A). Mega-scramblers were not used in any of the experimental procedures due to their rarity within the stock populations [46]. After collection, mites were housed individually (to avoid cannibalism and mating) in sealed, ventilated plastic tubes (50 mm high, 16 mm in diameter) that were filled up to three-fourths with a mix of plaster of Paris and charcoal powder for visual contrast. The plaster mix was made in batches by adding 40 ml tap water to 40 g plaster powder and one-third of a tablespoon of charcoal powder. The plaster was left to harden in the tubes for at least 24 h at room temperature before the tubes were used. The tubes were closed off with a cap containing a small air hole and a piece of fine mesh to prevent escaping while still allowing air flow. Each tube was hydrated—before the mites were placed inside—by adding two drops of tap water on the dried plaster layer with a drip pipette. After the mites were collected, one yeast granule was added to the tubes that housed mites from the rich stock populations, and one-fourth of an oat grain was added to the tubes that housed mites from the poor stock populations. Food was added in limited quantities to prevent mold growth on uneaten leftovers. All collected mites were kept in their individual tubes until they were used in pooled pheromone extractions (see below for details)—usually this constituted a period of 2–4 days in the tubes.

#### Body size measurements

The idiosoma length (the length of the body without mouthparts; Fig. 1A) of the collected mites was measured as a proxy for body size [42, 43]. First, the mites were photographed using a ZEISS Axiocam 105 color camera at 0.63–5 × magnification that was connected to a Zeiss Stemi 2000-C stereomicroscope. From these photos, the idiosoma length was measured to the nearest µm using Zen lite (Blue edition) analysis software. After enough individuals were measured (see below for details), mites from the same stock population that were of the same sex or ART were pooled for pheromone extractions based on similar idiosoma length—such that the variance in idiosoma length was as low as possible within each pool. The pooling of multiple mites was done to ensure quantifiable amounts of pheromone could be extracted (see Additional file 1). In total, 60 pools were created from measured mites (10 pools of females (n = 8–11), 10 pools of fighters (n = 10–14), and 10 pools of scramblers (n = 10–14) from the rich and the poor stock populations).

#### Pheromone extraction and gas chromatography (GC) analysis

Mites selected for pooled extractions were removed from their individual tubes and submerged together for 30 (± 1) minutes inside a screw-top glass vial filled with 50 µl of hexane containing 200 ng of pentadecane as the internal standard. The hexane extracts were then separated from the mites using a 100 µl Hamilton syringe, and stored in crimp-top vials at − 20 °C until further use. All pheromone extractions were performed within 30 h of the body size measurements. Once all extractions were completed, the extracts were prepared for gas chromatography (GC) analysis; extracts were evaporated down to 1–3 µl under a gentle nitrogen stream (at room temperature) and topped by ~ 1 µl of octane to prevent further evaporation. After preparation, the extracts were injected into a splitless inlet of a HP6890 GC coupled with a high resolution polar capillary column (DB‐WAXetr [extended temperature range]; 30 m × 0.25 mm × 0.5 µm) and a flame ionization detector (FID). The extracts were analyzed in three consecutive GC runs within a span of 72 h. Finally, α-acaridial quantities were calculated through integration of the putative α-acaridial peaks. Integration results were corrected by the differential response of the FID to the standards in each extract. Because the number of mites differed between pooled extractions, pheromone quantities of each extract were divided by the number of mites in that extract, to get the average per mite for each pool.

#### Gas chromatography-mass spectrometry (GC–MS) analysis

To confirm the presence of α-acaridial in the hexane extracts of the mites, gas chromatography-mass spectrometry (GC–MS) analysis was performed with three pooled extracts of females from poor stock populations. The extracts used for this analysis were obtained in the same manner as the extracts used for the GC analysis. The GC–MS analysis was performed using a Thermo Trace 1300 GC and Thermo Exactive Orbitrap MS (50 to 550 m/z scan range) operated at 70 eV in a splitless mode, with a DB5-MS capillary column (30 m × 0.25 mm × 0.25 µm). Helium was used as the carrier gas, and was delivered at 1.0 ml/min. The temperature was programmed to increase from 50 °C (1.5 min hold) to 320 °C at a rate of 5 °C /min.

### Statistical analysis

Using linear regression models, we analyzed the effects of morph (female, fighter or scrambler) (*Mo*), diet (rich or poor) (*D*) and idiosoma length (μm) (*IL*), and all their interactions, on log(α-acaridial production) (log ng) as the response variable *y* —α-acaridial production was log-transformed because the residuals of the model that we ran on the untransformed α-acaridial production values significantly differed from a Normal distribution (Shapiro test: W = 0.94; p = 0.007); but after log transformation, the model residuals did not differ from a Normal distribution (Shapiro test: W = 0.97, p = 0.29). The full statistical model was: *y = D + IL + Mo + D·IL + D·Mo + IL·Mo + D·IL·Mo*. The assumption of a Normal distribution of residuals was further justified by visual inspection of histograms of model residuals and normal quantile–quantile plots. The assumption of homogeneity of residuals was justified by visual inspection of residuals-versus-fits plots. We identified one female pool from the rich diet as an outlier (Grubbs test for one outlier, G = 4.99, U = 0.54, p < 0.001), and we had missing values resulting from unclear α-acaridial peaks for two fighter pools and two scrambler pools from the poor diet. Because of this unbalanced data structure, and the fact that we tested for interactive treatment effects, we used type III sums of squares. Finally, we assessed that there was no multicollinearity between the factors morph, diet and idiosoma length because their variance inflation factors were all between 1 and 5 (morph: 1.33, diet: 1.50, idiosoma length: 2.03).

To identify significant treatment effects, we used a stepwise, model simplification procedure whereby the full model was fitted, after which the least significant term was removed (starting with the highest order interaction) in case this deletion caused an insignificant increase in deviance [82]. Because each model simplification step involved a comparison between simpler model that was nested within a more complex model, we assessed significance by performing an F-ratio test [83]: *F* = [(*RSS*_*0*_* − RSS*_*1*_)/(*dƒ*_*0*_* − dƒ*_*1*_)]/(*RSS*_*1*_*/dƒ*_*1*_), where *RSS*_*i*_ and *df*_*i*_ are the residual sum of squares and degrees of freedom, respectively, of model *i* (where *i* = 0 as the simpler model and *i* = 1 as the more complex model). Non-significant *p*-values (*p* > 0.05) indicate that removing the term from the more complex model did not significantly increase the unexplained variance (residual deviance), and thus that the effect of the term on the response variable is non-significant. This procedure was repeated until the model only contained significant terms (p < 0.05). It turned out that this minimal model contained the factor morph, which has three levels (female, scrambler, fighter). To assess which of the different levels of the factor morph did not significantly different from each other, we merged different, pairwise combinations of the three different levels. For example, if a model where the morph levels fighter and scrambler are merged into one level ‘males’ does not result in a significant increase in residual deviance compared to the model where morph has three levels, inference is that fighters and scramblers do not significantly differ in log (α-acaridial production). All statistical analyses were conducted in R 4.1 [84].

## Supplementary Information

**Additional file 1:** Pilot hexane extractions.

## Data Availability

The datasets generated and/or analysed during the current study are available in the Figshare repository via https://doi.org/10.6084/m9.figshare.14842551.

## Abbreviations

ART: Alternative reproductive tactic
FID: Flame ionization detector
GC: Gas chromatography
GC–MS: Gas chromatography-mass spectrometry

## References

[1] Kodric-Brown A, Brown JH (1984). Truth in advertising: the kinds of traits favored by sexual selection. Am Nat.

[2] Oliveira RF, Taborsky M, Brockmann HJ (2008). Alternative reproductive tactics: an integrative approach.

[3] Brockmann HJ, Grafen A, Dawkins R (1979). Evolutionarily stable nesting strategy in a digger wasp. J Theor Biol.

[4] Gross MR (1991). Salmon breeding behavior and life history evolution in changing environments. Ecology.

[5] Bass A (1992). Dimorphic male brains and alternative reproductive tactics in a vocalizing fish. Trends Neurosci.

[6] Sinervo B, Lively CM (1996). The rock–paper–scissors game and the evolution of alternative male strategies. Nature.

[7] Emlen DJ (1997). Alternative reproductive tactics and male-dimorphism in the horned beetle Onthophagus acuminatus (Coleoptera: Scarabaeidae). Behav Ecol Sociobiol.

[8] Stewart KA, Hudson CM, Lougheed SC (2017). Can alternative mating tactics facilitate introgression across a hybrid zone by circumventing female choice?. J Evol Biol.

[9] Gadgil M (1972). Male dimorphism as a consequence of sexual selection. Am Nat.

[10] Emlen DJ (1994). Environmental control of horn length dimorphism in the beetle Onthophagus acuminatus (Coleoptera: Scarabaeidae). Proc Biol Sci.

[11] Tomkins JL (1999). Environmental and genetic determinants of the male forceps length dimorphism in the European earwig Forficula auricularia L. Behav Ecol Sociobiol.

[12] Cremer S, Heinze J (2003). Stress grows wings: environmental induction of winged dispersal males in Cardiocondyla ants. Curr Biol.

[13] Moczek AP, Hunt J, Emlen DJ, Simmons LW (2002). Threshold evolution in exotic populations of a polyphenic beetle. Evol Ecol Res.

[14] Piché J, Hutchings JA, Blanchard W (2008). Genetic variation in threshold reaction norms for alternative reproductive tactics in male Atlantic salmon, Salmo salar. Proc Biol Sci.

[15] Nettle D, Bateson M (1812). Adaptive developmental plasticity: what is it, how can we recognize it and when can it evolve?. Proc Biol Sci.

[16] Emlen DJ (1996). Artificial selection on horn length-body size allometry in the horned beetle Onthophagus acuminatus (Coleoptera: Scarabaeidae). Evolution.

[17] Smallegange IM (2011). Complex environmental effects on the expression of alternative reproductive phenotypes in the bulb mite. Evol Ecol.

[18] Rhebergen FT, Stewart KA, Smallegange IM. Nutrient-dependent allometric plasticity in a male-diphenic mite [Internet]. 2021. 10.1101/2021.06.14.448383. 10.1002/ece3.9145 PMC9343935 35928796

[19] Smallegange IM, Rhebergen FT, Stewart KA (2019). Cross-level considerations for explaining selection pressures and the maintenance of genetic variation in condition-dependent male morphs. Curr Opin Insect Sci.

[20] Godin J-GJ (1995). Predation risk and alternative mating tactics in male Trinidadian guppies (Poecilia reticulata). Oecologia.

[21] Shuster SM (1992). The reproductive behaviour of ɑ-, β-, Ɣ- male morphs in the *Paracerceis sculpta*, a marine isopod crustacean. Behaviour.

[22] Jukema J, Piersma T (2006). Permanent female mimics in a lekking shorebird. Biol Lett.

[23] Shine R, Phillips B, Waye H, LeMaster M, Mason RT (2001). Benefits of female mimicry in snakes. Nature.

[24] Shuster SM, Wade MJ (1991). Equal mating success among male reproductive strategies in a marine isopod. Nature.

[25] Norman MD, Finn J, Tregenza T (1999). Female impersonation as an alternative reproductive strategy in giant cuttlefish. Proc Biol Sci.

[26] Karlson P, Butenandt A (1959). Pheromones (Ectohormones) in insects. Annu Rev Entomol.

[27] Law JH, Regnier FE (1971). Pheromones. Annu Rev Biochem.

[28] Gomez-Diaz C, Benton R (2013). The joy of sex pheromones. EMBO Rep.

[29] De Pasqual C, Groot AT, Mappes J, Burdfield-Steel E (2021). Evolutionary importance of intraspecific variation in sex pheromones. Trends Ecol Evol.

[30] Ono T (1993). Effect of rearing temperature on pheromone component ratio in potato tuberworm moth, Phthorimaea operculella, (Lepidoptera: Gelechiidae. J Chem Ecol.

[31] Hock V, Chouinard G, Lucas E, Cormier D, Leskey T, Wright S (2014). Establishing abiotic and biotic factors necessary for reliable male pheromone production and attraction to pheromones by female plum curculios Conotrachelus nenuphar (Coleoptera: Curculionidae. Can Entomol.

[32] Boppré M, Schneider D (1985). Pyrrolizidine alkaloids quantitatively regulate both scent organ morphogenesis and pheromone biosynthesis in male Creatonotos moths (Lepidoptera: Arctiidae. J Comp Physiol A.

[33] Martín J, López P (2006). Vitamin D supplementation increases the attractiveness of males’ scent for female Iberian rock lizards. Proc Biol Sci.

[34] Edde PA, Phillips TW, Robertson JB, Dillwith JW (2007). Pheromone output by Rhyzopertha dominica (Coleoptera: Bostrichidae), as affected by host plant and beetle size. Ann Entomol Soc Am.

[35] Ming Q-L, Lewis SM (2010). Pheromone production by male Tribolium castaneum (Coleoptera: Tenebrionidae) is influenced by diet quality. J Econ Entomol.

[36] Weddle CB, Mitchell C, Bay SK, Sakaluk SK, Hunt J (2012). Sex-specific genotype-by-environment interactions for cuticular hydrocarbon expression in decorated crickets, Gryllodes sigillatus: implications for the evolution of signal reliability. J Evol Biol.

[37] Liedo P, Orozco D, Cruz-López L, Quintero JL, Becerra-Pérez C, del Refugio HM (2013). Effect of post-teneral diets on the performance of sterile *Anastrepha ludens* and *Anastrepha obliqua* fruit flies. J Appl Entomol.

[38] Blaul B, Steinbauer R, Merkl P, Merkl R, Tschochner H, Ruther J (2014). Oleic acid is a precursor of linoleic acid and the male sex pheromone in *Nasonia vitripennis*. Insect Biochem Mol Biol.

[39] Jensen K, Shearman M, Rapkin J, Carey MR, House CM, Hunt J (2017). Change in sex pheromone expression by nutritional shift in male cockroaches. Behav Ecol.

[40] Radwan J (1995). Male morph determination in two species of acarid mites. Heredity (Edinb).

[41] Deere JA, Smallegange IM (2014). Does frequency-dependence determine male morph survival in the bulb mite Rhizoglyphus robini?. Exp Appl Acarol.

[42] Fan QH, Zhang ZQ. Revision of Rhizoglyphus Claparède. Acari: Acaridae of Australasia and Oceania. Syst Appl Acarol Soc. 2004.

[43] Beuken TP, Duinmeijer CC, Smallegange IM (2019). Costs of weaponry: unarmed males sire more offspring than armed males in a male-dimorphic mite. J Evol Biol.

[44] Croll JC, Egas M, Smallegange IM (2019). An eco-evolutionary feedback loop between population dynamics and fighter expression affects the evolution of alternative reproductive tactics. J Anim Ecol.

[45] Radwan J, Czyz M, Konior M, Kolodziejczyk M (2000). Aggressiveness in two male morphs of the bulb mite Rhizoglyphus robini. Ethology.

[46] Stewart KA, Van den Beuken TPG, Rhebergen FT, Deere JA, Smallegange IM (2018). Evidence for a third male type in a male-dimorphic model species. Ecology.

[47] Smallegange IM, Coulson T (2011). The stochastic demography of two coexisting male morphs. Ecology.

[48] Smallegange IM, Thorne N, Charalambous M (2012). Fitness trade-offs and the maintenance of alternative male morphs in the bulb mite (Rhizoglyphus robini): fitness trade-offs in alternative male morphs. J Evol Biol.

[49] Radwan J (2003). Heritability of male morph in the bulb mite, Rhizoglyphus robini (Astigmata, Acaridae). Exp Appl Acarol.

[50] Stewart KA, Draaijer R, Kolasa MR, Smallegange IM (2019). The role of genetic diversity in the evolution and maintenance of environmentally-cued, male alternative reproductive tactics. BMC Evol Biol.

[51] Plesnar-Bielak A, Jawor A, Kramarz PE (2013). Complex response in size-related traits of bulb mites (Rhizoglyphus robini) under elevated thermal conditions - an experimental evolution approach. J Exp Biol.

[52] Radwan J, Klimas M (2001). Male dimorphism in the bulb mite, Rhizoglyphus robini: fighters survive better. Ethol Ecol Evol.

[53] Beuken TP, Smallegange IM (2018). Male nutritional history affects female fecundity in a male-dimorphic mite: evidence for a nuptial gift?. Evol Ecol.

[54] Gerson U, Cohen E, Capua S (1991). Bulb mite, Rhizoglyphus robini (Astigmata: Acaridae) as an experimental animal. Exp Appl Acarol.

[55] Leal WS, Kuwahara Y, Nakano Y, Nakao H, Suzuki T. A novel monoterpene from the acarid mite Tyrophagm perniciosm Acarina, Acaridae. Agric Biol Chem. 1989;2(E).

[56] Mizoguchi A, Mori N, Nishida R, Kuwahara Y (2003). α-Acaridial a female sex pheromone from an alarm pheromone emitting mite Rhizoglyphus robini. J Chem Ecol.

[57] Henneken J, Goodger JQD, Jones TM, Elgar MA (2017). Diet-mediated pheromones and signature mixtures can enforce signal reliability. Front Ecol Evol [Internet]..

[58] Brückner A, Heethoff M (2017). The ontogeny of oil gland chemistry in the oribatid mite *Archegozetes longisetosus* Aoki (Oribatida, Trhypochthoniidae). Int J Acarology.

[59] Pureswaran DS, Borden JH (2003). Is bigger better? Size and pheromone production in the Mountain Pine beetle, Dendroctonus ponderosae Hopkins (Coleoptera: Scolytidae). J Insect Behav.

[60] Byers JA (2005). A cost of alarm pheromone production in cotton aphids. Aphis gossypii Sci Nat.

[61] Ruther J, Matschke M, Garbe L-A, Steiner S (2009). Quantity matters: male sex pheromone signals mate quality in the parasitic wasp *Nasonia vitripennis*. Proc Biol Sci.

[62] Harari AR, Zahavi T, Thiéry D (2011). Fitness cost of pheromone production in signaling female moths: cost of pheromone production in moths. Evolution.

[63] Birgersson G, Schlyter F, Bergström G, Löfqvist J (1988). Individual variation in aggregation pheromone content of the bark beetle, Ips typographus. J Chem Ecol.

[64] Pankewitz F, Hilker M (2008). Polyketides in insects: ecological role of these widespread chemicals and evolutionary aspects of their biogenesis. Biol Rev Camb Philos Soc.

[65] Kuwahara Y, Shibata C, Akimoto K, Kuwahara M, Suzuki T (1988). Pheromone study on acarid mites. XIII. Identification of neryl formate as an alarm pheromone from the bulb mite, Rhizoglyphus robini Acarina: Acaridae. Appl Entomol Zool.

[66] Howard RW, Kuwahara Y, Suzuki H, Suzuki T (1988). Pheromone study on acarid mites. XII. Characterization of the hydrocarbons and external gland morphology of the opishonotal glands of six species of mites Acari: Astigmata. Appl Entomol Zool.

[67] Leal WS, Kuwahara Y, Suzuki T (1990). Robinal, a highly conjugated monoterpenoid from the mite Rhizoglyphus robini. Chemical ecology of astigmatid mites, XXVII (1). Naturwiss.

[68] Kuwahara Y, Cardé RT, Millar JG (2004). Chemical ecology of astigmatid mites. Advances in insect chemical ecology.

[69] Heethoff M, Raspotnig G (2012). Expanding the “enemy-free space” for oribatid mites: evidence for chemical defense of juvenile *Archegozetes longisetosus* against the rove beetle *Stenus juno*. Exp Appl Acarol.

[70] Barata EN, Serrano RM, Miranda A, Nogueira R, Hubbard PC, Canário AVM (2008). Putative pheromones from the anal glands of male blennies attract females and enhance male reproductive success. Anim Behav.

[71] Mazzoldi C, Rasotto MB (2002). Alternative male mating tactics in *Gobius niger*. J Fish Biol.

[72] Locatello L, Mazzoldi C, Rasotto MB (2002). Ejaculate of sneaker males is pheromonally inconspicuous in the black goby, *Gobius niger* (Teleostei, Gobiidae). J Exp Zool.

[73] Shumate AM, Teale SA, Ayres BD, Ayres MP (2011). Disruptive selection maintains variable pheromone blends in the bark beetle Ips pini. Environ Entomol.

[74] Collins RD, Cardé RT (1985). Variation in and heritability of aspects of pheromone production in the pink bollworm moth, Pectinophora gossypiella (Lepidoptera: Gelechiidae). Ann Entomol Soc Am.

[75] Zhu J, Chastain BB, Spohn BG, Haynes KF (1997). Assortative mating in two pheromone strains of the cabbage looper moth. Trichoplusia ni J Insect Behav.

[76] Brooks R, Hunt J, Blows MW, Smith MJ, Bussière LF, Jennions MD (2005). Experimental evidence for multivariate stabilizing sexual selection. Evolution.

[77] Smadja C, Butlin RK (2009). On the scent of speciation: the chemosensory system and its role in premating isolation. Heredity (Edinb).

[78] Groot AT, Schöfl G, Inglis O, Donnerhacke S, Classen A, Schmalz A (2014). Within-population variability in a moth sex pheromone blend: genetic basis and behavioural consequences. Proc Biol Sci.

[79] Groot AT, van Wijk M, Villacis-Perez E, Kuperus P, Schöfl G, van Veldhuizen D (2019). Within-population variability in a moth sex pheromone blend, part 2: selection towards fixation. R Soc Open Sci..

[80] Gerson U, Capua S, Thorens D (1983). Life history and life tables of Rhizoglyphus robini Claparède (Acari: Astigmata: Acaridae. Acarologia.

[81] Díaz A, Okabe K, Eckenrode CJ, Villani MG, Oconnor BM (2000). Biology, ecology, and management of the bulb mites of the genus Rhizoglyphus (Acari: Acaridae). Exp Appl Acarol.

[82] Crawley MJ (2012). The R book.

[83] Montgomery DC (1996). Design and analysis of experiments.

[84] R Core Team (2020). R: A language and environment for statistical computing. R Foundation for Statistical Computing, Vienna, Austria. https://www.R-project.org/.

