# Supplementary material for: Toward an understanding of the chemical ecology of alternative reproductive tactics in the bulb mite (Rhizoglyphus robini)
Source: BMC Ecol Evol. 2022 Jan 8;22:5. doi: 10.1186/s12862-021-01956-w (PMC8742560; doi:10.1186/s12862-021-01956-w)
Supplement: Supplementary file 1 — Additional file 1: Pilot hexane extractions. [file 12862_2021_1956_MOESM1_ESM.docx]

# **SUPPLEMENTARY INFORMATION: pilot hexane extractions**

To ensure quantifiable amounts of pheromone could be obtained from bulb mite hexane extracts, pilot extractions were performed. Individual mites and groups of mites, ranging from two to ten individuals, were submerged in various amounts of hexane for different durations (Table S1). All mites used in these extractions were randomly sampled from the stock populations, following the procedure described in the Methods. The extracts were analyzed through gas chromatography (GC), also following the procedure described in the Methods. Pilot extractions were deemed successful when clear, quantifiable pheromone peaks were seen in the resulting chromatograms. The results indicated that at least two females or ten males (mostly performed using fighters) were required to consistently obtain measurable amounts of pheromone from a single extract. Two additional pilot extractions were performed to check for potential contamination of yeast granules and oat grains (Table S1, bottom rows). This was done because food particles from the plastic tubes that housed the mites were sometimes accidentally submerged in the hexane along with the mites during the extractions. A yeast granule and an oat grain were individually submerged in 50 µl of hexane for 30 minutes. The resulting chromatographs did not contain notable peaks, indicating that these food particles would not contaminate mite extractions.

**Table S1:** Overview of the pilot extractions. The number of mites in each extraction, the sex and ART (for males), diet, amount of hexane used in the extraction and the extraction time (i.e., how long the mites were submerged in hexane) are given. The final column indicates whether the extractions resulted in quantifiable pheromone peaks.

| **Number of mites** | **Sex and ART** | **Diet** | **Hexane in extract (µl)** | **Extraction time (minutes)** | **Clear pheromone peak in chromatogram** |
| --- | --- | --- | --- | --- | --- |
| 1 | Female | Rich | 10 | 3 | No |
| 1 | Female | Rich | 50 | 10 | No |
| 8 | Female | Rich | 50 | 10 | Yes |
| 10 | Female | Poor | 50 | 10 | Yes |
| 4 | Female | Rich | 50 | 30 | Yes |
| 3 | Female | Rich | 50 | 30 | Yes |
| 2 | Female | Rich | 50 | 30 | Yes |
| 1 | Female | Rich | 50 | 30 | No |
| 1 | Fighter | Rich | 50 | 30 | No |
| 2 | Fighter | Rich | 50 | 30 | No |
| 3 | Fighter | Rich | 50 | 30 | No |
| 3 | Scrambler | Poor | 50 | 30 | No |
| 5 | Fighter | Rich | 50 | 30 | No |
| 10 | Fighter | Rich | 50 | 30 | Yes |
| Yeast granule |  |  | 50 | 30 | No |
| Oat grain |  |  | 50 | 30 | No |
